# UAV Enhanced Target-Barrier Coverage Algorithm for Wireless Sensor Networks Based on Reinforcement Learning

**DOI:** 10.3390/s22176381

**Published:** 2022-08-24

**Authors:** Li Li, Hongbin Chen

**Affiliations:** School of Information and Communication, Guilin University of Electronic Technology, Guilin 541004, China

**Keywords:** wireless sensor networks (WSNs), target-barrier coverage, Unmanned Aerial Vehicle (UAV), trajectory planning, reinforcement learning

## Abstract

Target-barrier coverage is a newly proposed coverage problem in wireless sensor networks (WSNs). The target-barrier is a closed barrier with a distance constraint from the target, which can detect intrusions from outside. In some applications, detecting intrusions from outside and monitoring the targets inside the barrier is necessary. However, due to the distance constraint, the target-barrier fails to monitor and detect the target breaching from inside in a timely manner. In this paper, we propose a convex hull attraction (CHA) algorithm to construct the target-barrier and a UAV-enhanced coverage (QUEC) algorithm based on reinforcement learning to cover targets. The CHA algorithm first divides the targets into clusters, then constructs the target-barrier for the outermost targets of the clusters, and the redundant sensors replace the failed sensors. Finally, the UAV’s path is planned based on QUEC. The UAV always covers the target, which is most likely to breach. The simulation results show that, compared with the target-barrier construction algorithm (TBC) and the virtual force algorithm (VFA), CHA can reduce the number of sensors required to construct the target-barrier and extend the target-barrier lifetime. Compared with the traveling salesman problem (TSP), QUEC can reduce the UAV’s coverage completion time, improve the energy efficiency of UAV and the efficiency of detecting targets breaching from inside.

## 1. Introduction

In recent years, UAVs have played a crucial role in sensor networks, and UAV-aided wireless sensor networks can significantly improve coverage [1]. The rise of UAV-aided wireless sensor networks has brought new opportunities for many applications, such as agriculture [2], environmental monitoring [3], data collection [4,5], animal detection [6], etc. Generally, coverage in WSNs can be classified into target coverage, area coverage, and barrier coverage [7]. Selecting different coverage types for different applications can significantly reduce the cost of WSNs. This paper mainly studies barrier coverage which can detect intrusions. There have been many studies on barrier coverage, which can be classified into the open and closed barrier. The open barrier is defined as constructing a continuous barrier that extends from one side to the opposite side. It fails in forming in an end-to-end fashion and can only detect intrusions from one side. Conversely, the closed barrier connects the head to the end of the barrier and forms a ring that can detect intrusions from any direction.

It is extremely critical to timely detect abnormal situations in some applications, such as wildlife monitoring, epidemic area monitoring, oil leak monitoring, etc. For example, in a wildlife monitoring scenario, it is necessary to detect intrusions from the outside to prevent humans from entering by accident or intruding illegally. At the same time, it is essential to monitor wild animals to detect the animals leaving their habitat or being in an unusual situation in time. The applications mentioned above must deploy a closed barrier with a distance constraint between the barrier and targets, and targets inside the closed barrier need to be monitored. Obviously, the open barrier and closed barrier cannot meet these requirements. The open barrier fails to detect intrusions from any direction, and the closed barrier without the distance constraint between the barrier and targets cannot detect intrusions from outside in advance. The authors of [8] proposed a target-barrier coverage type, which forms a continuous closed barrier around the target, and has a distance constraint between the barrier and targets. In [9], a deterministic deployment algorithm is proposed to study how to construct the target-barrier with the minimum number of sensors. Based on [9], reference [10] applied the target-barrier to agriculture. UAVs are used as mobile nodes and hover to form a closed barrier. When agricultural pests approach the target-barrier, UAVs quickly land on the ground to kill them. All the above-mentioned studies have achieved good results, which will be described in the next section. Nevertheless, there are still some shortcomings, as follows:

- Many existing target-barrier coverage algorithms assume that the sensors are static. A large number of sensors need to be deployed to guarantee the construction of the target-barrier successfully, and the cost is high.
- Network lifetime is an essential parameter of WSNs. However, most studies do not consider the target-barrier lifetime.
- Although the existing target-barrier coverage algorithms can detect intrusion from outside in time, in most cases, the distance constraint is so large that the target-barrier cannot detect targets breaching from the inside effectively.

In this paper, we propose a convex hull attraction algorithm (CHA) and a reinforcement learning-based UAV enhanced coverage algorithm (QUEC). The target-barrier is constructed with a smaller number of sensors, and the target-barrier lifetime can be prolonged by moving the sensors appropriately. Simultaneously, the UAV flies to cover the targets to detect them breaching from inside in time. The main contributions of this paper are summarized as follows.

(1) We explicitly consider the cost of constructing the target-barrier and the target-barrier lifetime, and the CHA algorithm is proposed. We divide the targets into clusters and construct the target-barrier for each cluster’s outermost targets, making it unnecessary to construct the target-barrier for each target. Then, the redundant sensors are moved to replace the failed sensors in the target-barrier. Through the above-mentioned methods, the number of sensors required to construct the target-barrier can be greatly reduced, and the target-barrier lifetime can be prolonged.
(2) In this paper, we additionally consider the coverage of targets inside the target-barrier and propose a QUEC algorithm. To the best of our knowledge, this is the first study to detect target breaching from inside the target-barrier. The UAV’s path is optimized based on reinforcement learning, and the reward and punishment mechanism of reinforcement learning are applied to allow the UAV to autonomously choose the targets to cover. The UAV always covers the target, which is likely to breach from the inside, to detect targets breaching from inside in time.

The rest of this paper is organized as follows. Section 2 briefly introduces some related works about barrier coverage, UAV, and reinforcement learning. Section 3 describes the models and problem formulations. Section 4 presents the proposed CHA and QUEC for forming target-barrier and planning the UAV’s path in detail. A performance evaluation of the proposed algorithms is given in Section 5. Finally, Section 6 gives some conclusions.

## 2. Related Works

Barrier coverage can be further classified into open barrier coverage, closed barrier coverage, and target-barrier coverage. In this section, the related works on the barrier coverage problem of deployment and the planning of the UAV’s path are presented.

Open barrier coverage [11,12,13,14,15,16,17,18,19]: The authors of [11] pointed out the limitations of the barrier coverage algorithms based on global information and developed a barrier coverage protocol based on local information (LBCP). LBCP can not only guarantee local barrier coverage but also prolong the network lifetime. The authors of [12] investigated the barrier coverage problem of a bi-static radar (BR) sensor network and proposed that the optimal coverage can be achieved by adjusting the placement order and spacing of BRs. In [13], a barrier coverage algorithm based on the environment pareto-dominated selection strategy is proposed for the coverage problem of multi-constraint sensor networks, which can improve the coverage ratio effectively. In [14], barrier coverage was applied to the traffic count, and two coverage mechanisms, weighted-based working scheduling (WBWS) and connectivity-based working scheduling (CBWS) were proposed. WBWS and CBWS can significantly reduce the number of nodes required while guaranteeing user-defined surveillance quality. Reference [15] proposed an efficient *k*-barrier coverage mechanism. The number of nodes required to construct a *k*-barrier is reduced by developing the cover adjacent net, and a barrier energy scheduling is proposed to achieve its energy balance. Reference [16] considered the barrier coverage problem in rechargeable sensor networks based on the probability sensing model and proposed a barrier coverage algorithm called MCDP. The MCDP calculates the detection probability of each sensor to each space time point and schedules sensors to stay in the sensing state and charging state in each time slot. The authors of [17] proposed a coordinated sensor patrolling (CSP) algorithm, which exploits the information about intruder arrivals in the past to guide each sensor’s movement. An efficient distributed deployment algorithm was proposed to enhance barrier coverage in [18]. The proposed algorithm can reduce the communication energy consumption and the moving distance of sensors. Reference [19] pointed out that by deploying the sink stations in a mobile sensor network, the number of sensors required to construct a barrier can be known, and the moving distance of sensors can be optimized. The above-mentioned barrier coverage algorithms form the open barrier which cannot detect intrusion from any direction.

Closed barrier coverage [20,21,22,23]: Reference [20] proposed an algorithm based on virtual force to form the closed barrier surrounding the region. This algorithm cannot directly be applied to construct the target-barrier, because it determines the boundary by sensing the targets and has no distance constraint between the barrier and targets. In [21], it is assumed that each sensor can cover an angle, and the 360° coverage can be achieved by finding multiple coverage sets. Reference [22] proposed a software-defined system consisting of the cloud-based architecture and the barrier maintenance algorithm to control the movement of each sensor in real-time to form the barrier surrounding a dynamic zone adaptively. In [23], multiple multimedia sensors were deployed to form several cover sets, and each cover set can form a closed barrier in the region of interest. The cover sets were scheduled to be activated serially to prolong the network lifetime.

Target-barrier coverage [8,9,10,24]: Reference [8] proposed a target-barrier coverage algorithm. Four sensors in four directions that are nearest to the targets and satisfy the distance constraint were selected, and the remaining members were selected based on the smallest angle with the start sensor and destination sensor. Then, the intersecting barriers were merged into a barrier to reduce the number of sensors required. However, the static sensors cannot move, and a large number of sensors are required to guarantee that the target-barrier can be constructed successfully. Reference [9] proposed a deterministic deployment algorithm to form the target-barrier and exploited the merging property to reduce the number of sensors required. Although the algorithm can greatly reduce the number of sensors required, it is not flexible. Based on [10], the author proposed replacing static sensors with UAVs. The UAVs are served as mobile nodes and hover to form the target-barrier. Although the mobile nodes are applied, the role of the mobile nodes is only for hovering and landing [10]. The directional sensors were deployed to construct the target-barrier by rotating the orientation of the sensors [24]. However, this algorithm cannot be used directly for omnidirectional sensors.

The above-mentioned algorithms can effectively detect intrusions from outside. Nevertheless, covering the targets and detecting them breaching from inside is not considered. Compared with mobile sensors, ground unmanned vehicles, and mobile robots, UAVs that can fly at a higher altitude and have better flexibility are widely used [25,26]. UAVs are usually used for area coverage [27] and target coverage [28] to collect information. On the other hand, UAVs are also frequently used to assist in task offloading [29], security authentication [30], trust evaluation [31], etc. Reference [27] investigated the effects of UAV mobility patterns on area coverage. Reference [28] proposed a weighted targets sweep coverage (WTSC) algorithm. Although WTSC can improve the coverage quality and shorten the UAV’s flight time, the trajectory cannot be adjusted in a timely manner when the environment changes. In the Internet of Vehicles system under the 6G network, UAVs are to provide a task offloading platform for devices to offload tasks [29]. The authors of [30] pointed out that UAVs are a flexible solution to the infrastructure-less vehicular networks for secure authentication and key management. In [31], the UAV is sent to collect the code wait to be verified from bedrock devices to evaluate the trust of the mobile vehicles (MVs) to prevent malicious MVs from disseminating the code to the sensing devices.

When the number of UAVs is fixed, it is the key to optimizing the flight trajectory to improve the coverage quality and reduce energy consumption. There are many meta-heuristic approaches for planning UAV’s paths, such as the ant colony optimization algorithm [32], particle swarm optimization algorithm [33,34], genetic algorithm [35], etc. Reinforcement learning is also applied to plan the path [36,37,38,39]. In [36], a UAV serves as an aerial base station for ground users. The UAV can intelligently track ground users without knowing the user-side information and channel parameters based on reinforcement learning. Reference [37] proposed a UAV’s path planning algorithm based on reinforcement learning, and the UAV can successfully avoid obstacles and provide coverage for targets. The authors of [38] proposed to use the UAVs as the relay nodes for forwarding signals and a source node for sending signals and proposed a multi-objective path optimization method based on Q-learning to adapt to the dynamic changes of the network. In [39], the UAVs were applied to offload tasks from the user equipment, and a trajectory planning algorithm based on reinforcement learning was proposed to adapt to the changes in the environment. In summary, the UAV can better adapt to the environment based on reinforcement learning. Therefore, the UAV’s path was planned based on reinforcement learning in this paper, and the UAV can learn the target breaching from inside and cover the target that is the most likely to breach.

## 3. Models and Problem Formulations

In this section, the network environment and assumptions in UAV-assisted wireless sensor networks are introduced first. Then, the models and problem formulations are given.

### 3.1. Wsn Model

*N* mobile sensors are randomly deployed in the area, we use the *S* set to represent the sensors, $S=\{S_{1},S_{2},\ldots ,S_{N}\}$, and the coordinates of the sensor $S_{i}$ are denoted by $\left(x_{S}^{i},y_{S}^{i}\right)$(i=1,2,…,N). It is assumed that there are *M* targets in the area. *T* represents the set of targets, that is $T=\{T_{1},T_{2},\ldots ,T_{M}\}$, the coordinates of target $T_{j}$ are $\left(x_{T}^{j},y_{T}^{j}\right)$(j=1,2,…,M). The WSN consists of the working sensors and redundant sensors.

**Figure 1:**
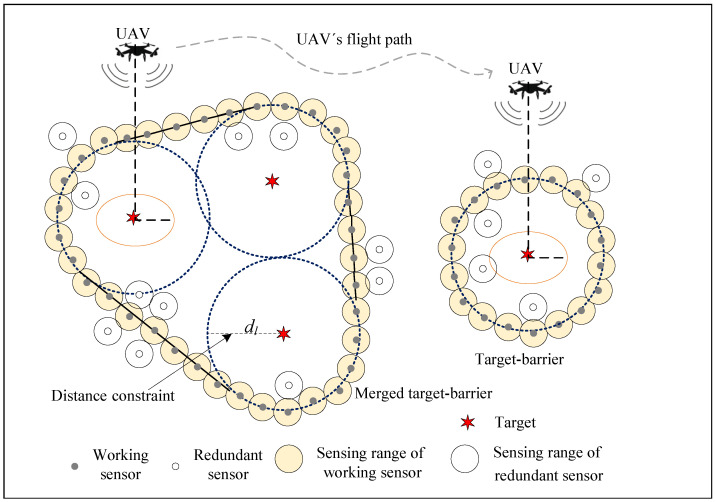
WSN model.

All the sensors are relocated after being deployed randomly. After the initial relocation, some sensors are used to form the target-barrier. They are marked as working sensors to detect intrusions. The remaining sensors are marked as redundant and sleeping after the initial relocation to reduce energy consumption. When the energy of the working sensors is about to be exhausted, they will be replaced by redundant sensors. In addition, a single UAV serves as an aerial node to cover the targets to detect if they breach from inside in time. Assume that the transmission radius of the UAV is *R*, the flight altitude is *H*, and the UAV ground coverage radius is $R_{UAV}$ = $\sqrt{R^{2}-H^{2}}$. The UAV’s altitude cannot be too high to ensure that the UAV can cover the ground targets, H<R. To maximize the coverage of the UAV, the lower the UAV’s altitude, the better. However, the UAV’s altitude cannot be too low for safety. Therefore, we fixed the UAV’s altitude at the minimum safe size. The WSN model is shown in Figure 1.

To simplify the problem analysis, our discussion is based on the following assumptions:

(1) Each sensor knows its location through GPS or localization algorithm [8]. The position of the targets and the distance constraint $d_{l}$ are stored in the memory of the sensors before deployment.
(2) All sensors are homogeneous and have the same initial energy. The sensing radius is $R_{S}$, and the communication radius of the sensor is $R_{C}$.
(3) The number of sensors required to form the target-barrier cannot be known in advance, so it is assumed that the density of sensors is suitable, and the sensors are redundant.

**Definition** **1.** *(Target-barrier coverage [8]): A target-barrier is constructed in a closed barrier around the target. There is a constraint between the target and target-barrier, which depends on applications and needs.*

**Definition** **2.** *(Target-barrier lifetime): The target-barrier lifetime is the period from when the target-barrier starts to work until it cannot work. In this paper, the target-barrier lifetime is measured by the number of rounds for which the sensors can make the target-barrier work normally.*

### 3.2. Target-Barrier Coverage Model

The CHA algorithm first divides the targets into clusters according to the locations of targets and $d_{l}$, then moves the sensors to form the target-barrier. Because there is a distance constraint between the target barrier and the target, it can detect intrusion from any direction in advance. In some extreme cases, if the targets’ locations change, they would be informed to the sensors through the UAV. Additionally, the sensors would move to form the new target-barrier.

### 3.3. Target Breaching Detection Model

The UAV always covers the target that most likely breaches from inside each time. In this paper, each target is assigned a weight indicating its importance. The greater the weight, the more likely the target is to breach from inside. Furthermore, the weight will change as the environment changes. It is noted as *W*, $W\in \left[0,W_{th}\right]$. Note that the weight of target *m* at the time slot *t* is $W_{t}^{m}$. If $W_{t}^{m}<W_{th}$, it means that the target is safe and it will not breach from inside. Otherwise, the target will start breaching from inside. To simplify the analysis, we assume the weight change ratio is $W_{ave}$, and the time required to detect target breaching from inside is denoted as $t=\left(W_{th}-W_{t}^{m}\right)/W_{ave}$.

### 3.4. Energy Consumption Model

#### 3.4.1. The Energy Consumption of Sensors

In this paper, the sensors move to form the target-barrier and perform the sensing task. Therefore, we mainly consider the energy consumption of the sensors as moving energy consumption and sensing energy consumption. A sensor’s moving energy consumption is $E_{move}=e*d_{move}$, where *e* is the energy consumption of the sensor as it moves 1 m, and $d_{move}$ is the distance that the sensor moves. The sensing energy consumption of a sensor to perform the sensing task in a round is proportional to $R_{S}^{2}$ or $R_{S}^{4}$ [40]. The sensing energy consumption in a round is $E_{S}$, and the sensing energy consumption model adopted in this paper is $E_{S}=\beta *R_{S}^{2}$, where β is the coefficient.

#### 3.4.2. The Energy Consumption of UAV

Assuming the UAV flies at a fixed altitude *H* and a constant speed $V_{u}$. Divide the total working time of the UAV into *T* time slots, and the location of the UAV at the time slot *t* is $q_{t}=\left(x_{UAV}^{t},y_{UAV}^{t},H\right)$. We consider only the energy consumed by the flight and hover power and do not include the transmission power. This paper ignores the acceleration and deceleration during flying, and the flight power is regarded as a constant. The flight power is $P_{f}$, and the energy efficiency of traveling 1 m can be defined as $e=P_{f}/V_{u}$ [41]. The energy consumption of the UAV for the flight distance $d_{f}$ can be expressed as $E_{f}=e*d_{f}$. The hover power is $P_{h}$, the hovering time of the UAV above the target *m* is $T_{m}^{h}$. Therefore, the total energy consumption of the UAV is $E_{t}=E_{f}+P_{h}*T_{m}^{h}$.

### 3.5. Problem Formulations

#### 3.5.1. Target-Barrier Coverage

When the energy of the working sensor reaches the energy threshold, the redundant sensor would replace the working sensor to prolong the target-barrier lifetime. The fewer sensors that construct the target-barrier, the more redundant sensors can replace the working sensors, and the longer the target-barrier lifetime is. Therefore, the optimization problem is transformed into the problem of how to form the target-barrier with fewer sensors. The optimization problem is formulated as follows:

(1) $$\begin{array}{cc} min\; & \left[\sum_{i=1}^{h}|S\left(B_{h}\right)|\right] \end{array}$$

subject to

(2) $$\begin{array}{cc} \; & d\left(S_{i},T_{j}\right)\geq d_{l},\forall S_{i}\in S\left(B_{h}\right),T_{j}\in T\left(B_{h}\right),B_{h}\in B \end{array}$$

(3) $$\begin{array}{cc} \; & d\left(S_{i},S_{i+1}\right)\leq 2*R_{S},\forall S_{i}\in S\left(B_{h}\right),B_{h}\in B \end{array}$$

(4) $$\begin{array}{cc} \; & \sum_{i=1}^{h}T\left(B_{h}\right)=M,\forall B_{h}\in B \end{array}$$

where *B* is the set of the target-barriers formed, $B=\{B_{1},B_{2},\ldots ,B_{h}\}$, $S(B_{h})$ is the set of the sensors contained in the target-barrier $B_{h}$, $T(B_{h})$ is the set of targets surrounded by the target-barrier $B_{h}$, $d_{l}$ is the distance constraint between the target-barrier and targets, and $d(S_{i},T_{j})$ is the distance between the sensor contained in the target-barrier and target. Constraint (2) imposes that the distance between the sensors contained in the target-barrier and the targets is not less than $d_{l}$. Constraint (3) guarantees that the sensing regions of sensors overlap with each other in the target-barrier. Constraint (4) shows that all targets should be surrounded by the target-barrier.

#### 3.5.2. UAV-Assisted Target-Barrier Coverage

The UAV is applied to assist in covering and detecting. When planning the UAV’s path, it is hoped that the UAV covers the target with the largest weight and completes the tasks of coverage and detection with lower energy consumption. Therefore, the optimization problem can be transformed into maximizing the ratio of weights to energy consumption.

(5) $$\begin{array}{c} max\;\theta \end{array}$$

subject to

(6) $$\begin{array}{cc} \; & {\left(x_{t}^{n}-x_{t-1}^{n}\right)}^{2}+{\left(y_{t}^{n}-y_{t-1}^{n}\right)}^{2}\leq V_{u}^{2},\forall t=1,2,\ldots ,T \end{array}$$

where $\theta =\sum_{t\in T}\sum_{m\in M}W_{t}^{m}/E_{t}$.

## 4. Algorithm Descriptions

### 4.1. Target-Barrier Coverage Algorithm

When the distance between two targets is not greater than $2d_{l}$, placing them on the same target-barrier can reduce the number of sensors required to form the target-barrier [8]. The CHA algorithm firstly merges the targets that meet the requirement in the same cluster and constructs target-barrier for only the outermost targets. Through [9,20], we know that the shortest perimeter enclosing the region is its convex hull. When the target-barrier constructed is a convex hull, it can significantly reduce the number of sensors required. It is assumed that the convex hull is attractive. Under the attraction of the convex hull and the attraction and repulsion between sensors, some sensors would move until they are uniformly distributed on the convex hull of the region. The steps of CHA are shown in Algorithm 1, as follows.
**Algorithm 1** Implementation of CHA Algorithm.**Input:** $T,d_{l}$, Sensor’s initial energy $E_{0}$, round, $R_{S}$, $E_{S}$**Output:** Target-barrier set with minimum sensors $S(B_{h})$ and round  1:   Choose a target $T_{j}$ from *T*, $B_{h}=T_{h}⋃B_{h}$, $T=T-T_{j}$  2:   While T≠∅  3:   Find all the targets whose distance from $T_{j}$ is not greater than $2d_{l}$, add to $B_{h}$, and remove from *T*;  4:   Select the target from $B_{h}$, repeat 2–3, until no targets can be found from *T* whose distance to any target in $B_{h}$ is not greater than $2d_{l}$. In this case, $B_{h}$ is a cluster;  5:   Repeat 1–4 until T=∅, and all clusters are obtained;  6:   end while  7:   Find the outermost targets of all clusters;  8:   Some sensors are moved to the convex hull and marked as working sensors, the set of working sensors is $S(B_{h})$. Besides, the remaining sensors are moved to surround the convex hull, and they are marked as redundant sensors and in sleep;  9:   Calculate the remaining energy after all sensors have moved;10:   While (1)11:   The energy consumed by the working sensor in a round is $E_{S}$, update $E_{0}=E_{0}-E_{S}$, and round=round+1;12:   if the number of working sensors with residual energy less than $E_{S}$ is greater than the number of redundant sensors;13:      break14:   else15:           Find the redundant sensors with the shortest distance from the working sensors, and the redundant sensors replace the working sensors;16:   end if17:   end while

### 4.2. Uav Trajectory Optimization

In this section, the UAV’s path is optimized to cover the target and detect it breaching from inside in time. We hope the UAV can learn the target breaching and then automatically choose the target to cover. The trajectory of the UAV can be regarded as a Markov decision process (MDP), and the Q-learning deals with the trajectory of the UAV. We define state by S, action by A, and reward by R. At each time slot, the agent observes the state s∈S of the current environment and selects an action a∈A based on the current state and the experience learned in the past. Then the agent receives a reward *r* and transitions to a new state $s^{\prime }\in S$ according to the transition probability $P[S_{t+1}=s^{\prime },R_{t+1}=r|S_{t}=s,A_{t}=a]$. The state, action, and reward defined in this section are as follows.

State: When dividing the monitoring area into multiple grids of equal size, the size of the monitoring area and the distance between the targets need to be considered. The size of the grid divided here is $size=\lambda \times \lfloor d_{min}\rfloor$, where λ=1/2 is the proportion parameter, and $d_{min}$ is the minimum distance between the targets. The rows are rowsize=w/size, where *w* is the width of the monitoring area. At the time slot *t*, the horizontal coordinates of the UAV are $\left(x_{UAV}^{t},y_{UAV}^{t}\right)$, the corresponding coordinates of the grid are $\left(g,h\right)=(\left\lceil x_{UAV}^{t}/size\right\rceil ,\left\lceil y_{UAV}^{t}/size\right\rceil )$, and the state of the UAV is state=(g−1)×rowsize+h [42].

Action: The actions of the UAV are discrete into east, south, west, north, southeast, southwest, northwest, northeast, and hover. To better represent the state of UAV, the horizontal coordinate changes of UAV are size respectively.

Reward: The reward types of UAV are hovering and flying. (1) When the target is in the coverage area of the UAV, and the weight of the target does not reach the weight threshold, the UAV will be rewarded $r_{h}=1$ for hovering. Otherwise, the UAV will be punished $r_{nh}=-1$ for flying. (2) In each step, the inverse of the distance between the UAV and target is used as the reward for guiding the UAV to fly to the target, $r_{1}=-\xi \left(d\left(UAV,T_{j}\right)-R_{UAV}\right)$, where ξ=0.01 is the gain of reward, $d(UAV,T_{j})$ is the distance between the UAV and target, and $R_{UAV}$ is the coverage radius of the UAV. The closer the UAV is to the target after acting, the greater the reward will be obtained. Additionally, the reward is $r_{2}=\eta \left(d_{old}-d_{new}\right)$, where η=0.08 is the gain factor, $d_{old}$ is the distance between the UAV and target at the previous time slot, and $d_{new}$ is the distance between the UAV and target at the current time slot. We define a reward $r_{3}=2$ when the UAV successfully covers the target. Therefore, the total reward for each step of the UAV is r=r1+r2+r3. The steps of QUEC are as follows in Algorithm 2.
**Algorithm 2** Implementation of QUEC Algorithm.   Initialize action space and state space; set learning rate α, discount factor γ, exploration probability ϵ, and Q(s,a);   Maximum training episodes $N_{e}$; Maximum steps of each episode roundmax;   for $episode=1:N_{e}$      Initialize the state of the agent and the time step $n_{1}\leftarrow 0$; Calculate $t_{m}$;      while (1)         Select the action *a* based on ϵ−greedy;         Perform *a*, observe reward *r* and the next state $s^{\prime }$;         Update Q(s,a);         $Q\left(s,a\right)\leftarrow Q\left(s,a\right)+\alpha [R+\gamma *max_{a^{\prime }}Q\left(s^{\prime },a^{\prime }\right)-Q\left(s,a\right)]$;         Update $s^{\prime }\leftarrow s$;         Calculate and update $t_{m}$;         n←n+1;         if n>roundmax or or the set of the targets is empty;      end while   end for

## 5. Simulations

The performance of the proposed CHA is evaluated in this section. Specifically, we compare CHA with the target-barrier construction algorithm (TBC) [8] and the virtual force algorithm (VFA) [20]. The TBC, which studies similar coverage problems to ours, is the first algorithm proposed to solve the target-barrier coverage problem. The TBC is to construct a target-barrier for each target and then merge the intersecting barriers into a barrier. Furthermore, this paper considers that the sensors are movable after the initial deployment. Additionally, the VFA, as a classical algorithm, can move the sensors to the proper location. In [20], the VFA is used to construct the closed barrier on the boundary of a single monitoring area based on the virtual force and then to adjust the positions of the sensors so that the sensors are evenly distributed on the convex hull. It can automatically form the closed barrier. For the convenience of comparison, we integrate the merging mechanism into VFA. Since our proposed CHA includes merging the targets to form the clusters and construction of the closed barrier with the distance constraint, we compare TBC and VFA with CHA to prove the performance of CHA.

### 5.1. Simulation Environment

There are 10 targets randomly deployed in a 600 m × 600 m monitoring area. The targets are divided into a cluster by CHA, with 7 targets at the outermost of the cluster. The sensor’s sensing radius is 10 m, and the distance constraint $d_{l}$ is 80 m. Furthermore, the other parameters used in the simulations are shown in Table 1.

### 5.2. Simulation Results

**Figure 2:**
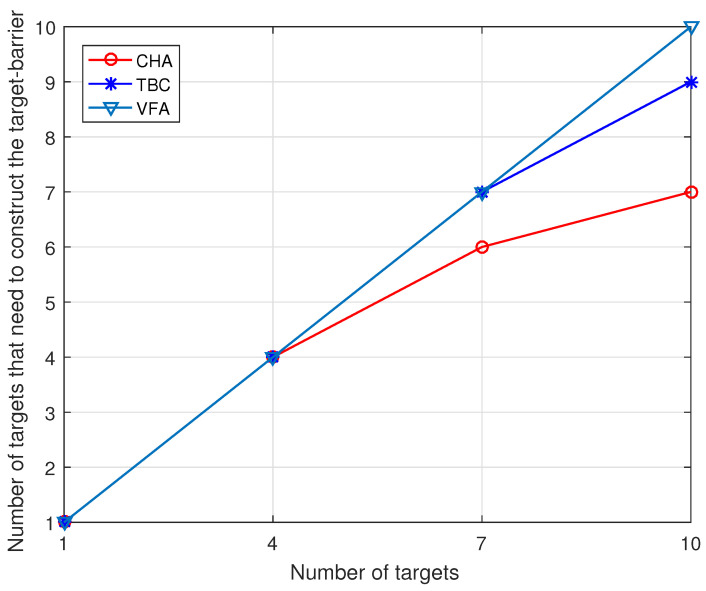
Number of targets that need to construct the target-barrier versus Number of targets.

**Figure 3:**
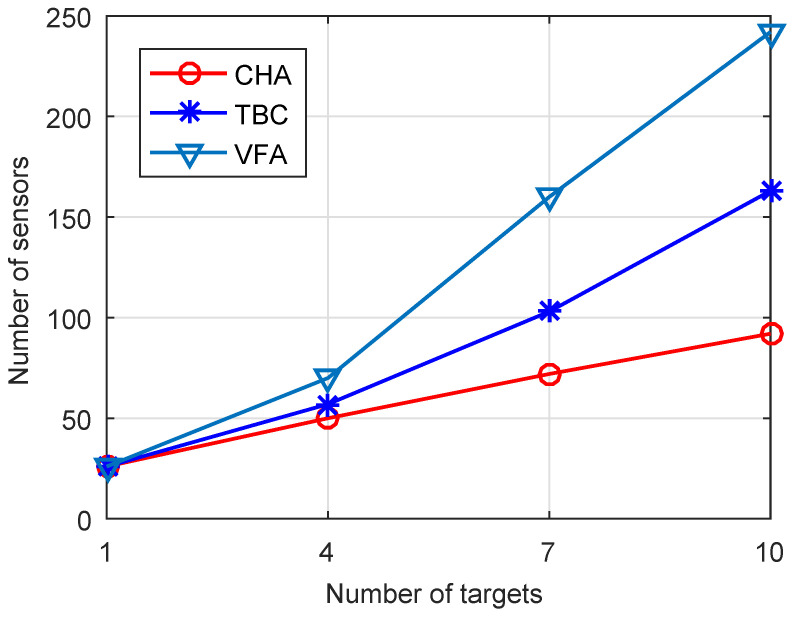
Numbers of sensors versus Number of targets.

In the first experiment, we explore the number of targets that need to construct the target-barrier under different target numbers, the number of sensors required to construct the target-barrier with varied numbers of targets, and the target-barrier lifetime with varied numbers of sensors when the number of targets is 10. As shown in Figure 2, when the number of targets is large, the CHA algorithm only needs to construct the target-barrier for some targets. The reason for this is that in the CHA algorithm, we first cluster the targets and then find the outermost targets of the cluster. We only need to construct the target-barrier for the outermost targets of the cluster to make all targets within the target-barrier. Figure 3 compares the number of sensors required to form the target-barrier with varied numbers of targets. As shown in this figure, the number of sensors required to form the target-barrier increases with the increase of targets. When the number of targets increases, the CHA algorithm needs fewer sensors to construct the target-barrier than the benchmark algorithms. The reason for this is that the CHA algorithm constructs the target-barrier only for the outermost targets. Additionally, the target-barrier is a convex hull, which can significantly reduce the sensors required to construct the target-barrier. In contrast, the benchmark algorithms construct the target-barrier for all targets.

**Figure 4:**
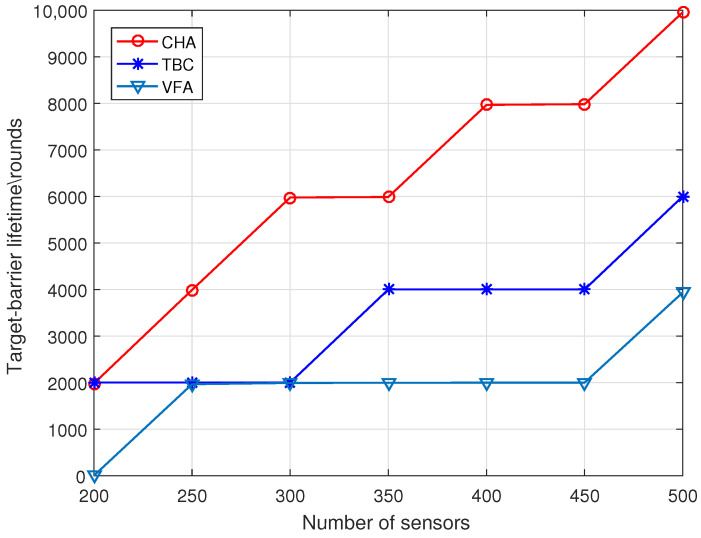
Target-barrier lifetime versus Number of sensors.

As shown in Figure 4, when the number of targets is 10, the three algorithms’ target-barrier lifetime increases with the number of sensors. However, the growth of the CHA algorithm is more significant than that of the benchmark algorithms, and the target-barrier lifetime of the CHA algorithm is much higher than that of the benchmark algorithms. The reason for this is that the number of sensors required by the CHA algorithm to construct the target-barrier is much less than that of the benchmark algorithms. In addition, the CHA algorithm can replace the failed working sensors by moving redundant sensors.

**Figure 5:**
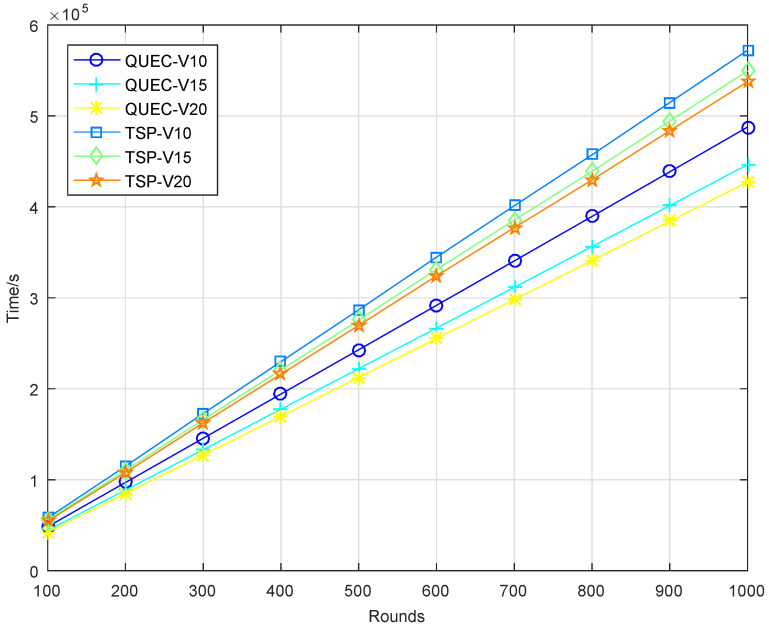
Time required to complete the coverage task.

**Figure 6:**
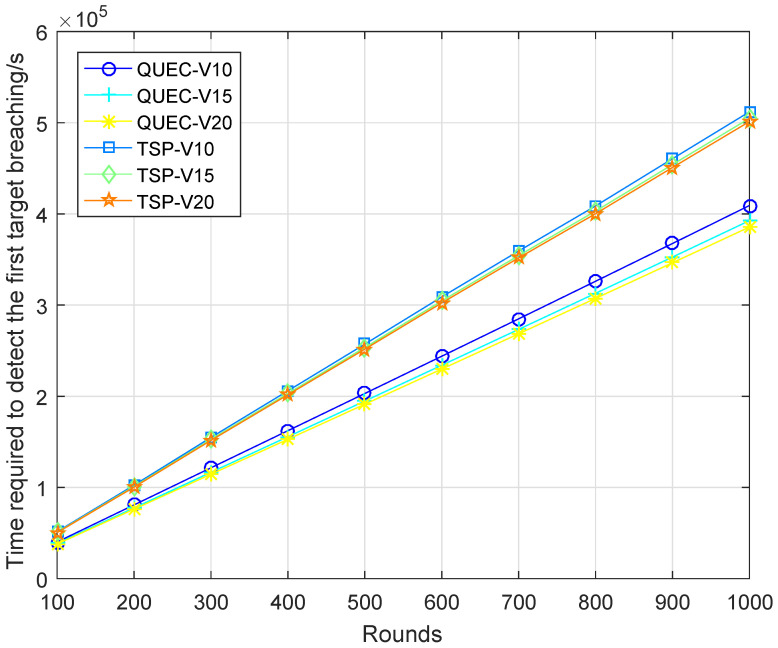
Time required to detect the first target breaching.

**Figure 7:**
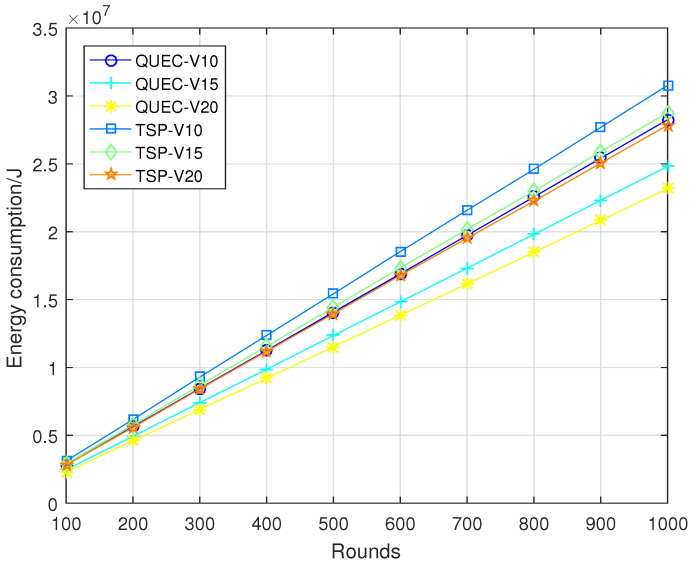
Energy consumption.

**Figure 8:**
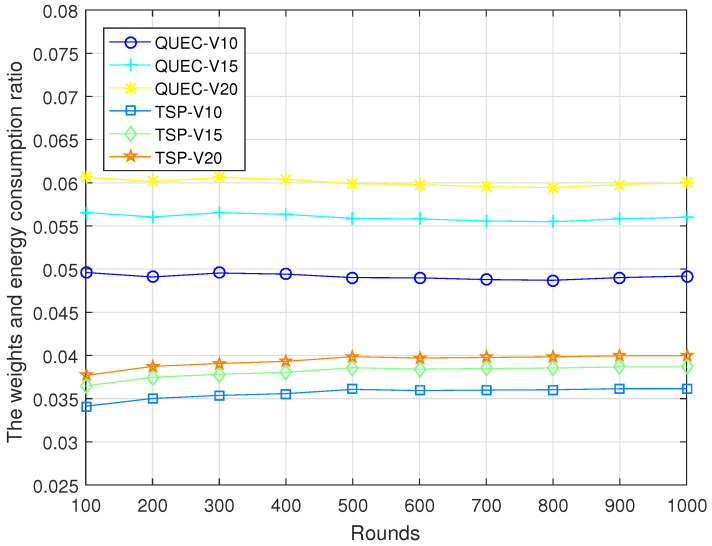
The weights and energy consumption ratio.

In the second experiment, completing the coverage mission means that the UAV detects all the targets breaching from inside, which is defined as a round. To compare the performance of the UAV with and without learning, we compare QUEC with the classic traveling salesman problem (TSP), which is solved based on the ant colony algorithm. The ant colony algorithm is to find the shortest flight path of the UAV, which was first introduced by Marco Dorigo in his Ph.D. thesis [43]. To test the performance of QUEC, we compared the time required for the UAV to complete the coverage task, the time required to detect the first target breaching from inside, the energy consumption of the UAV, and the ratio of the weights to the energy consumption at varied flight speeds, as shown in Figure 5, Figure 6, Figure 7 and Figure 8.

**Figure 9:**
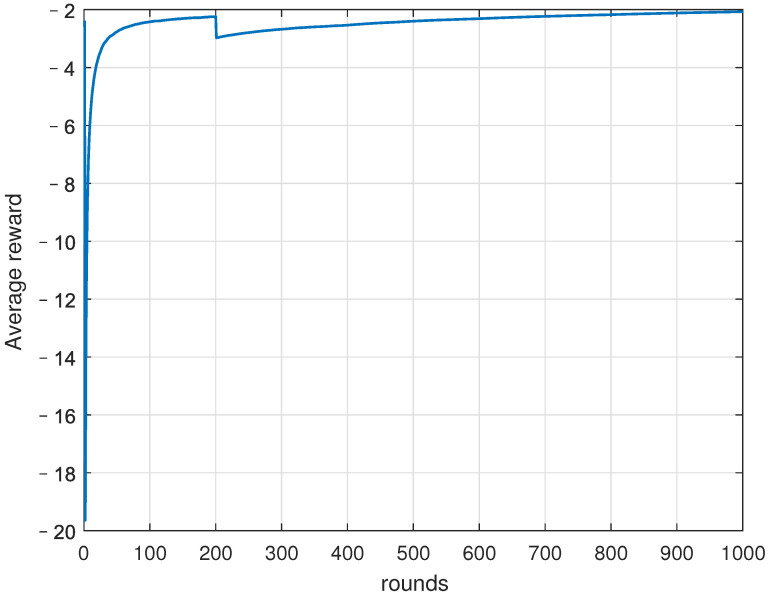
Average reward.

Figure 9 shows the average reward of the UAV during training. We can see that the proposed QUEC is convergent. Although the average reward received by the UAV fluctuates, it increases with learning rounds in general and tends to stabilize around the 800th round.

Figure 5 shows the time required for the UAV to complete the coverage task. As can be seen from the results, the time required for QUEC to complete the coverage task is shorter than that of TSP. For QUEC, it always makes the UAV fly towards the target with the largest weight and gives priority to covering the target that may breach from inside, which significantly reduces the time for the UAV to provide continuous coverage for the target. As shown in this figure, the time required to complete the coverage task decreases as the speed increases. The higher the speed, the shorter the flight time of the UAV is. This is a natural phenomenon. We can observe that QUEC takes less time to complete the coverage task at a speed of 10 m/s than TSP at a speed of 20 m/s, which further verifies the advantages of our proposed algorithm. Figure 6 shows that QUEC takes less time to detect the first target breaching from inside than TSP, and the time increases with the number of rounds. The UAV flies to cover the target with the largest weight first, so it can quickly detect the first target breaching. Furthermore, the time required for each coverage after that will be shorter than that of the TSP. It is worth noting that the line of TSP at a speed of 20 m/s is still higher than the line of QUEC at a speed of 10 m/s. This again verifies the advantage of QUEC.

Figure 7 shows the energy consumption of QUEC and TSP. We can observe that the proposed QUEC has less energy consumption than the benchmark algorithm. When the speed of the UAV is 10 m/s, the proposed QUEC reduces energy consumption by 8% compared to TSP. Moreover, when the speed of UAV is 20 m/s, the proposed QUEC reduces energy consumption by 17% compared to TSP. It is not particularly obvious that QUEC consumes less energy than TSP, especially when the speed of the UAV is 10 m/s. The reason for this is that energy consumption is related to distance. The UAV sometimes chooses a far away but most-weighted target to cover, and this will increase energy consumption to some extent. Figure 8 shows the ratio of the weights of the targets to the energy consumption of the UAV. As can be seen from Figure 8, the energy efficiency of the QUEC algorithm is higher than that of the TSP. This can verify the advantage of the proposed algorithm. The UAV always covers the target with the largest weight.

## 6. Conclusions

In this paper, the CHA and QUEC algorithms are proposed. The CHA algorithm is divided into three parts, clustering, constructing the target-barrier, and replacing the failed working sensors with redundant sensors. Additionally, the QUEC optimizes the trajectory of the UAV based on reinforcement learning to detect the target breaching from inside in time. Simulation results indicate that the scheme proposed in this paper can reduce the number of sensors required, prolong the lifetime of the target-barrier, and detect the targets breaching from the inside in time. However, when obstacles in the monitored area prevent the sensors from moving, the target barrier may have coverage holes. Furthermore, the coverage detection time for a single UAV may increase significantly in large-scale networks. Therefore, in the future, we will adjust the network model such as cooperating UAVs with ground sensors to construct the target-barrier, and focus on the cooperative coverage of UAV swarms to adapt to more complex scenarios.

## Figures and Tables

**Table 1:** Simulation parameters.

Parameters	Value
Monitoring area	600m×600m
Number of sensors *N*	200–500
Sensing radius $R_{s}$	10m
Distance constraint $d_{l}$	80m
Number of targets *M*	10
The sensor’s initial energy $E_{0}$	1J
The unit energy consumption of the sensor *e*	$5\times 10^{-5}\;J/m$
Proportion factor β	$5\times 10^{-6}\;\left(\mathrm{round}.J/m\right)$
The UAV’s altitude *H*	6m
The initial position of the UAV	[300,300, H]
The coverage radius of the UAV $R_{UAV}$	50m
The flying speed of the UAV	10 m/s–20 m/s
The flying power of the UAV $P_{f}$	70W
The hovering power of the UAV $P_{h}$	50W
Learning rate α	0.01
Discount factor γ	0.9
The threshold of weight $W_{th}$	350
The weight change ratio $W_{ave}$	2

## Data Availability

Not applicable.

## References

[1] Muhammad Y., Arafa H., Sangman M. (2020). Routing protocols for UAV-aided wireless sensor networks. Science.

[2] Lin C., Han G., Qi X., Du J., Xu T., Martínez-García M. (2021). Energy-optimal data collection for unmanned aerial vehicle-aided industrial wireless sensor network-based agricultural monitoring system: A clustering compressed sampling approach. IEEE Trans. Ind. Inform..

[3] Pan M., Chen C., Yin X., Huang Z. (2022). UAV-aided emergency environmental monitoring in infrastructure-less areas: LoRa mesh networking approach. IEEE Internet Things.

[4] Wang Y., Chen M., Pan C., Wang K., Pan Y. (2022). Joint optimization of UAV Trajectory and sensor uploading powers for UAV-assisted data collection in wireless sensor networks. IEEE Internet Things.

[5] Yoon I., Noh D. (2022). Adaptive data collection using UAV with wireless power transfer for wireless rechargeable sensor networks. IEEE Access.

[6] Gu J., Su T., Wang Q., Du X., Guizani M. (2018). Multiple moving targets surveillance based on a cooperative network for multi-UAV. IEEE Commun. Mag..

[7] Sharma R., Prakash S. Coverage problems in WSN: A survey and open issues. Proceedings of the 2018 Second International Conference on Intelligent Computing and Control Systems (ICICCS).

[8] Cheng C.-F., Wang C.-W. (2018). The target-barrier coverage problem in wireless sensor networks. IEEE Trans. Mob. Comput..

[9] Si P., Wang L., Shu R., Fu Z. (2021). Optimal deployment for target-barrier coverage problems in wireless sensor networks. IEEE Syst. J..

[10] Si P., Fu Z., Shu L., Yang Y., Huang K., Liu Y. (2022). Target-barrier coverage improvement in an insecticidal lamps internet of UAVs. IEEE Trans. Veh. Technol..

[11] Chen A., Kumar S., Lai T.H. (2009). Local barrier coverage in wireless sensor networks. IEEE Trans. Mob. Comput..

[12] Gong X., Zhang J., Cochran D., Xing K. (2016). Optimal placement for barrier coverage in bistatic radar sensor networks. IEEE/ACM Trans. Netw..

[13] Zhuang Y., Wu C., Zhang Y., Jia Z. (2017). Compound event barrier coverage algorithm based on environment pareto dominated selection strategy in multi-constraints sensor networks. IEEE Access.

[14] Chang C.-Y., Kuo Y.-W., Xu P., Chen H. (2018). Monitoring quality guaranteed barrier coverage mechanism for traffic counting in wireless sensor networks. IEEE Access.

[15] Weng C.-I., Chang C.-Y., Hsiao C.-Y., Chang C.-T., Chen H. (2018). On-Supporting energy balanced *k* -barrier coverage in wireless sensor networks. IEEE Access.

[16] Xu P., Wu J., Chang C.-Y., Shang C., Roy D.S. (2021). MCDP: Maximizing cooperative detection probability for barrier coverage in rechargeable wireless sensor networks. IEEE Sens. J..

[17] He S., Chen J., Li X., Shen X., Sun Y. (2014). Mobility and intruder prior information improving the barrier coverage of sparse sensor networks. IEEE Trans. Mob. Comput..

[18] Nguyen T.G., So-In C. (2018). Distributed Deployment algorithm for barrier coverage in mobile sensor networks. IEEE Access.

[19] Li S., Shen H., Huang Q., Guo L. (2019). Optimizing the Sensor movement for barrier coverage in a sink-based deployed mobile sensor network. IEEE Access.

[20] Kong L., Liu X., Li Z., Wu M.-Y. Automatic barrier coverage formation with mobile sensor networks. Proceedings of the 2010 IEEE International Conference on Communications.

[21] Hung K., Lui K. (2010). On perimeter coverage in wireless sensor networks. IEEE Trans. Wirel. Commun..

[22] Kong L., Lin S., Xie W., Qiao X., Jin X., Zeng P., Ren W., Liu X.-Y. (2016). Adaptive barrier coverage using software defined sensor networks. IEEE Sens. J..

[23] Lalama A., Khernane N., Mostefaoui A. Closed peripheral coverage in wireless multimedia sensor networks. Proceedings of the 15th ACM International Symposium on Mobility Management and Wireless Access.

[24] Chen G., Xiong Y., She J., Wu W., Galkowski K. (2021). Optimization of the directional sensor networks with rotatable sensors for target-barrier coverage. IEEE Sens. J..

[25] Sun P., Boukerche A., Tao Y. Theoretical analysis of the area coverage in a UAV-based wireless sensor network. Proceedings of the 2017 13th International Conference on Distributed Computing in Sensor Systems (DCOSS).

[26] Baek J., Han S.I., Han Y. (2019). Energy-efficient UAV routing for wireless sensor networks. IEEE Trans. Veh. Technol..

[27] Rashed S., Mujdat S. (2017). Analyzing the effects of UAV mobility patterns on data collection in wireless sensor networks. Sensors.

[28] Li J., Xiong Y., She J., Wu W. (2020). A path planning method for sweep coverage with multiple UAVs. IEEE Internet Things.

[29] Liu R., Liu A., Qu Z., Xiong N. (2021). An UAV-enabled intelligent connected transportation system with 6G Communications for internet of vehicles. IEEE Trans. Intell. Transp..

[30] Tan H., Zheng W., Vijayakumar P., Sakurai K., Kumar N. (2022). An efficient vehicle-assisted aggregate authentication scheme for infrastructure-less vehicular networks. IEEE Trans. Intell. Transp..

[31] Liang J., Liu W., Xiong N., Liu A., Zhang S. (2022). An intelligent and trust UAV-assisted code dissemination 5G system for industrial internet-of-things. IEEE Trans. Ind. Inform..

[32] Zhen Z., Chen Y., Wen L., Han B. (2020). An intelligent cooperative mission planning scheme of UAV swarm in uncertain dynamic environment. Aerosp. Sci. Technol..

[33] Sanchez-Garcia J., Reina D.G., Toral S.L. (2018). A distributed PSO-based exploration algorithm for a UAV network assisting a disaster scenario. Future Gener. Comp. Syst..

[34] Huang C., Fei J., Deng W. (2020). A Novel route planning method of fixed-wing unmanned aerial vehicle based on improved QPSO. IEEE Access.

[35] Pehlivanoglu Y.V., Pehlivanoglu P. (2021). An enhanced genetic algorithm for path planning of autonomous UAV in target coverage problems. Appl. Soft. Comput..

[36] Yin S., Zhao S., Zhao Y., Yu F.R. (2019). Intelligent trajectory design in UAV-aided communications with reinforcement learning. IEEE Trans. Veh. Technol..

[37] Li Y., Zhang S., Ye F., Jiang T., Li Y. A UAV path planning method based on deep reinforcement learning. Proceedings of the 2020 IEEE USNC-CNC-URSI North American Radio Science Meeting (Joint with AP-S Symposium).

[38] Liu J., Wang Q., He C., Jaffrès K., Xu Y., Li Z., Xu Y. (2020). QMR: Q-learning based multi-objective optimization routing protocol for flying ad hoc networks. Comput. Commun..

[39] Wang L., Wang K., Pan C., Xu W., Aslam N., Nallanathan A. (2021). Deep reinforcement learning based dynamic trajectory control for UAV-assisted mobile edge computing. IEEE Trans. Mob. Comput..

[40] Wu J., Yang S. Coverage issue in sensor networks with adjustable ranges. Proceedings of the international Conference on Parallel Processing Workshops.

[41] Arafat M., Moh S. (2022). JRCS: Joint routing and charging strategy for logistics drones. IEEE Internet Things.

[42] Konar A., Goswami Chakraborty I., Singh S.J., Jain L.C., Nagar A.K. (2013). A deterministic improved Q-learning for path planning of a mobile robot. IEEE Trans. Syst. Man Cybern..

[43] Dewantoro R.W., Sihombing P., Sutarman. The combination of Ant Colony Optimization (ACO) and Tabu Search (TS) algorithm to solve the Traveling Salesman Problem (TSP). Proceedings of the 2019 3rd International Conference on Electrical, Telecommunication and Computer Engineering (ELTICOM).

